# Disrupted Ultradian Activity Rhythms and Differential Expression of Several Clock Genes in Interleukin-6-Deficient Mice

**DOI:** 10.3389/fneur.2017.00099

**Published:** 2017-03-22

**Authors:** Francisco J. Monje, Ana Cicvaric, Juan Pablo Acevedo Aguilar, Immanuel Elbau, Orsolya Horvath, Weifei Diao, Micaela Glat, Daniela D. Pollak

**Affiliations:** ^1^Department of Neurophysiology and Neuropharmacology, Center for Physiology and Pharmacology, Medical University of Vienna, Vienna, Austria; ^2^Max Planck Institute of Psychiatry, Munich, Germany

**Keywords:** interleukin 6, circadian activity, mouse, behavior, clock gene, hippocampus

## Abstract

The characteristics of the cycles of activity and rest stand out among the most intensively investigated aspects of circadian rhythmicity in humans and experimental animals. Alterations in the circadian patterns of activity and rest are strongly linked to cognitive and emotional dysfunctions in severe mental illnesses such as Alzheimer’s disease (AD) and major depression (MDD). The proinflammatory cytokine interleukin 6 (IL-6) has been prominently associated with the pathogenesis of AD and MDD. However, the potential involvement of IL-6 in the modulation of the diurnal rhythms of activity and rest has not been investigated. Here, we set out to study the role of IL-6 in circadian rhythmicity through the characterization of patterns of behavioral locomotor activity in IL-6 knockout (IL-6 KO) mice and wild-type littermate controls. Deletion of IL-6 did not alter the length of the circadian period or the amount of locomotor activity under either light-entrained or free-running conditions. IL-6 KO mice also presented a normal phase shift in response to light exposure at night. However, the temporal architecture of the behavioral rhythmicity throughout the day, as characterized by the quantity of ultradian activity bouts, was significantly impaired under light-entrained and free-running conditions in IL-6 KO. Moreover, the assessment of clock gene expression in the hippocampus, a brain region involved in AD and depression, revealed altered levels of *cry1, dec2*, and *rev-erb-beta* in IL-6 KO mice. These data propose that IL-6 participates in the regulation of ultradian activity/rest rhythmicity and clock gene expression in the mammalian brain. Furthermore, we propose IL-6-dependent circadian misalignment as a common pathogenetic principle in some neurodegenerative and neuropsychiatric disorders.

## Introduction

Changes in the diurnal oscillations of the periods of activity and rest are in the spotlight of basic and applied biomedical research on circadian rhythms in humans and other animals (1). The interest in analyzing these changes in active wakefulness and quiescent rest rhythmicity relates to the fact that alterations of these rhythmic fluctuations are associated with a wide spectrum of pathologies, ranging from metabolic and cardiovascular dysfunctions to tumorigenesis and cancer. In the neurosciences, the consequences of circadian disruptions and chronic misalignments have been most prominently studied with regards to their effects on cognitive and emotional functions within the framework of some of the most severe neurological and psychiatric illnesses. Specifically, strong clinical and experimental evidence supports a link between disturbances of the sleep–wake cycle and other physiological functions regulated by the circadian system in the pathophysiology of Alzheimer’s disease (AD) and major depression (MDD). These dysfunctions include interruptions of the wakefulness during the day and bursts of activity during the night in individuals suffering from AD (2–6).

In addition, it has been described that part of the clinical symptomatology in AD patients is exacerbated at particular periods of the day, most commonly in the early evening (2, 7–10). In addition, a derangement in the circadian rhythmicity of several physiological functions (including the regulation of body temperature and hormone release) is frequently observed (11–15).

Similarly, MDD patients often report disrupted sleep–wake cycles and impairments in the diurnal patterns of other physiological processes [as reviewed in Ref. (16)]. In parallel to the reported “sun downing” in AD, MDD patients often also show significant diurnal mood swings with depressive symptoms usually being strongest in the morning (1).

At the molecular level, polymorphisms and expressional changes in several clock genes, the genetic elements constituting the molecular machinery organizing endogenous circadian rhythmicity, have been identified in postmortem samples of AD and MDD patients and animal models thereof (15, 17–29). Together with the shared involvement of circadian disruptions, both MDD and AD have been associated with altered inflammatory states (30, 31). The pro-inflammatory cytokine interleukin 6 (IL-6) (32), which is linked to circadian clock-related inflammation (33), is considered to play a central role in the pathophysiology of MDD and AD (30, 31, 34–39). Indeed, IL-6 has been proposed as a molecular bridge between circadian and inflammatory processes in a chronobiological animal model of depression (40) and is implicated in circadian rhythmicity (41) and in the circadian regulation of sleep drive (42, 43). Moreover, its secretion is determined by a marked diurnal pattern (44–46), and several clock genes are known as regulator of its production (47, 48).

However, the specific relationship between IL-6 and the diurnal rhythms of activity and rest remain poorly understood as varying observations regarding IL-6 levels under physiological and pathology conditions emerge from literature. These apparent discrepancies may be a consequence of species-specific effects and/or depend on the sample type or methodological approaches employed (31, 44–46, 49). Hence, further investigations using specific, genetically engineered animals are warranted. We here, therefore, set out to examine the involvement of IL-6 in the regulation of behavioral circadian rhythms by studying the changes in the diurnal patterns of locomotor activity in constitutive IL-6 knockout mice (IL-6 KO) in comparison with their wild-type (WT) littermate controls. To determine the impact of IL-6 deletion on the orchestration of circadian rhythmicity at the molecular level, the expression of 19 clock and clock-controlled genes was analyzed in the hippocampus, a brain region importantly implicated in the pathophysiology of MDD and AD.

## Materials and Methods

### Animals

Experiments were carried out in male adult IL-6 KO (B6.129S2-Il6tm1Kopf/J) and WT littermate control mice (Jackson Laboratories, Bar Harbor, ME, USA) (*n* = 9–11 per group). All mice were 8- to 10-week old at the time of experiments. Mice were housed individually in Nalgene cages equipped with running wheels (15 cm in diameter; Actrimetrics, Evanston, IL, USA) in a sound-attenuated room with constant temperature of 22 ± 2°C. Before experimental assessment of the circadian activity all animals were kept on a light/dark (LD) cycle of 12:12 h with lights on at 6 a.m. and off at 6 p.m. During the light phase, mice were exposed to a light intensity of ~200 lux. During conditions of constant darkness [dark/dark (DD)] defined as LD cycle of 0:24 h, the cage cleaning and animal care taking was carried out under dim red light (15 W). Mice were supplied with food and tap water *ad libitum* throughout the experimental period. All experiments were designed to minimize animal suffering and the number of animals used. Animal procedures were approved by the Austrian ethical committee (BMWF-66.009/0069-II/36/2011) on animal care and use conducted in accordance with international laws and policies.

### Assessment of Circadian Wheel-Running Activity

#### Acquisition

**Figure 1:**
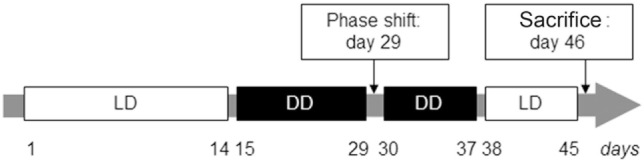
**Experimental paradigm for the evaluation of light-entrained and free-running circadian rhythms in interleukin (IL)-6 knockout (IL-6 KO) and wild-type (WT) mice**. Illustration of the temporal course (in days) for the analysis of circadian behavioral locomotor activity in IL-6 KO and WT mice under light-entrainment [light/dark (LD): 12 h light and 12 h dark phase; white boxes] and during settings of free-running rhythms [dark/dark (DD): 24 h constant darkness, black boxes].

Wheel revolutions were recorded using the ClockLab computer software, with sampling epochs of 1 min (Actimetrics, Evanston, IL, USA). After 1 week of habituation to the vivarium, the light-entrained daily activity was assessed for 14 days during LD followed by the evaluation of the free-running circadian activity during DD. On day 29, DD was briefly interrupted by a light pulse (30 min, 300 lux) at circadian time (CT) 16 (4 h after activity onset) for the induction of a phase-shift response to evaluate the response of the endogenous circadian pacemakers to external *zeitgebers*. After 7 additional days of DD, all mice were exposed to LD for 7 days before sacrifice on day 46 (Figure 1).

#### Analysis

Wheel-running activity was analyzed using the ClockLab software package (Actimetrics, Evanston, IL, USA) as previously described (27, 50). The default software settings were used to determine the activity onsets, which were manually edited when appropriate. Measures of the entrainment period (*T*) in LD and circadian period (tau) in DD and the total activity were derived from regression lines fit to the activity onsets. Activity bouts were defined as periods during which activity never reached less than 1 count per minute (bout threshold) for longer than 18 min (maximum gap length) at a time. All parameters were determined for each animal under LD and DD conditions. Phase-shift responses were evaluated by comparing the predicted activity onset for the day after light pulse treatment from extrapolated lines of the activity onsets of the days preceding the light pulse and 7 days after the pulse.

### Gene Expression Analysis

#### Brain Dissection

All brain dissections were carried out during the light phase of the circadian cycle (between 9 a.m. and 11 a.m.). Mice were sacrificed by neck dislocation, and brains were rapidly dissected over ice and total hippocampi were bilaterally collected and stored in RNA later^®^ (Ambion, Austria, Austin, TX, USA) at −20°C until used for RNA isolation or kept at −80°C for protein expression studies.

#### RNA Isolation, cDNA Synthesis, and Quantitative Real-time Polymerase Chain Reaction (qRT-PCR)

RNA was isolated from hippocampal tissues using the miRNeasy kit (Qiagen^®^, USA, Hilden, Germany) following the instructions of the manufacturer. Briefly, 900 ng of total RNA was used for cDNA synthesis using the MMLV reverse transcriptase first-strand cDNA synthesis kit G1 (Biozym^®^, Hessisch Oldendorf, Germany) following the manufacturer’s instructions. The resulting cDNA reaction mix (1:10 dilution) was used for PCR amplification using the Fast SYBR Green Mastermix (Applied Biosystems, Foster City, CA, USA) on a StepOnePlus real-time PCR system (serial no. 271000455; Applied Biosystems, Foster City, CA, USA). All reactions were carried out in duplicates. Primer sequences for all clock were analyzed: *brain and muscle aryl hydrocarbon receptor nuclear translocator-like 1* (*bmal1*), *circadian locomotor output cycles kaput* (*clock*), *cryptochrome 1/2* (*cry1/2*), *deleted in esophageal cancer 1/2* (*dec1/2*), *neuroD1, neuronal PAS domain-containing protein 2* (*npas2*), *period 1–3* (*per1–3*), *reverse erythroblastosis virus* α*/*β (*rev-erb*α*/*β) and *RAR* (*retinoic acid receptor*)*-related orphan receptor* α*-*γ (*ror*α*-*γ) and clock-controlled genes *D site of albumin promoter* (*albumin D-box*) *binding protein* (*dbp*), *E4 promoter-binding protein 4* (*e4bp4*), *inhibitor of DNA binding 2* (*id2*), and *neuronal differentiation 1* are listed in the Supplementary Table 1 of Ref. (27)

The C(*t*) values of β-actin were used for calculation of ΔC(*t*), representing the relative quantification of mRNA amounts in each sample. This further allowed the calculation of ΔΔC(*t*), subtracting mean ΔC(*t*) value of the WT from the mean ΔC(*t*) value for the KO. ΔΔC(*t*) was then used to express the fold change of mRNA levels observed between WT and KO mice, using the formula 2^−ΔΔC(t)^.

### Statistical Analysis

BioStat software (AnalystSoft Inc., Alexandria, VA, USA) was used for statistical analysis. Comparisons between two groups were determined using unpaired two-tailed Student’s *t*-test. In addition, two-way analysis of variance (ANOVA) (light condition × genotype) was employed for statistical evaluation of locomotor activity (alpha, rho, and total) and for bout analysis (number of bouts/day, bout length and counts/bout). The level of significance was set at *p* < 0.05 in all instances.

## Results

### IL-6 KO Mice Present with Fragmented Daily Activity Patterns under LD and DD Conditions

**Figure 2:**
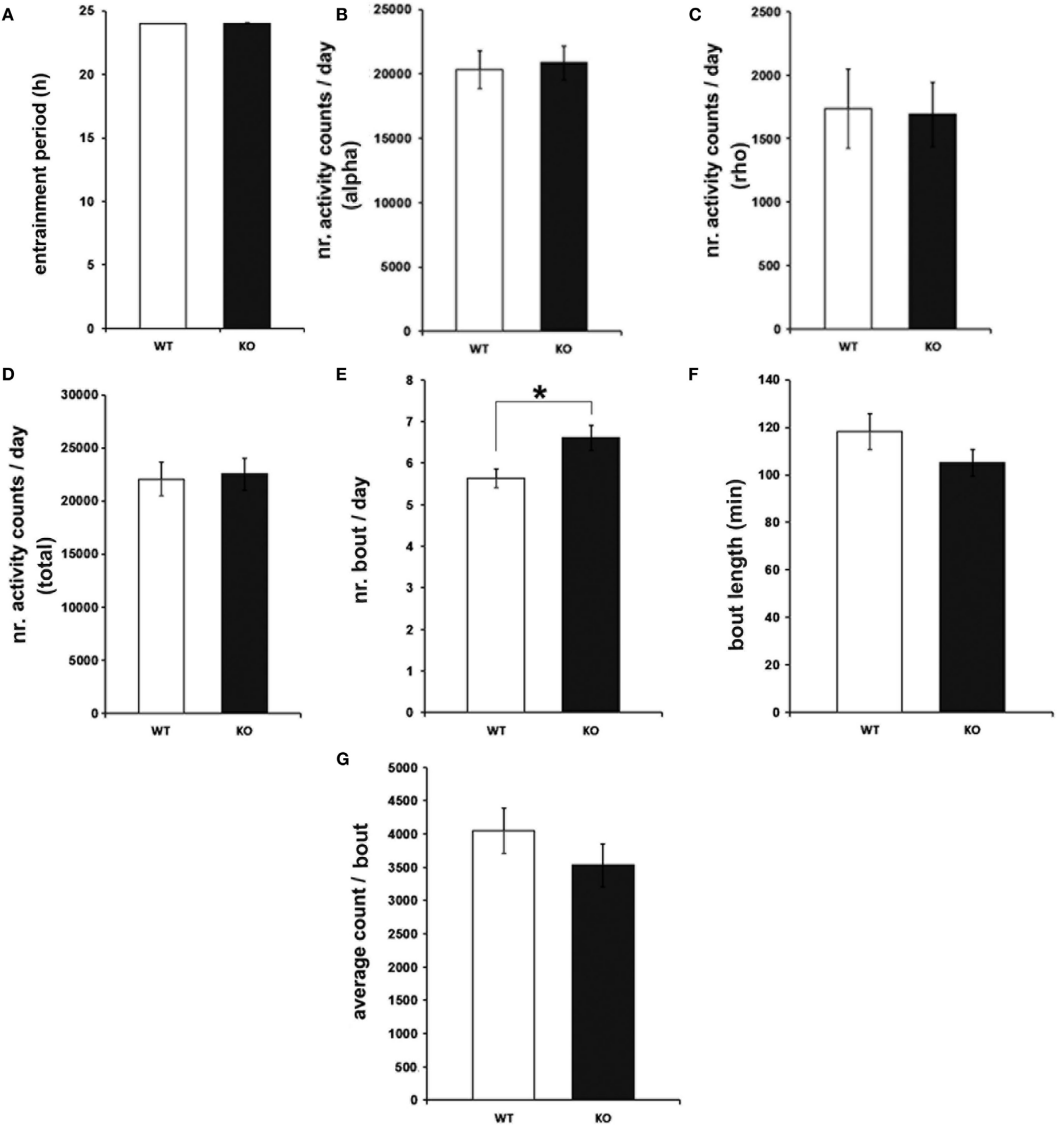
**Entrainment period (*T*), wheel-running activity, and bout analysis in under light-entrained [light/dark] conditions in interleukin (IL)-6 knockout (IL-6 KO) and wild-type (WT) mice**. Analysis of the light-entrained circadian behavioral locomotor activity in IL-6 KO and WT mice (*n* = 9–11 per group) demonstrating comparable **(A)** *T* and wheel-running activity during the **(B)** alpha and **(C)** rho phase and in **(D)** total amounts. **(E)** Significantly increased quantity of activity bouts in IL-6 KO compared with WT mice with unaltered **(F)** bout length and **(G)** activity counts/bout. All data are displayed as mean ± SEM; *p < 0.05.

To characterize the effects of genetic IL-6 deficiency on behavioral rhythms of rest and activity, wheel-running activity was monitored in IL-6 KO and WT littermate control mice. The investigation of light-entrained rhythms under LD conditions indicated unaltered length of the entrainment period (*T*) (Figure 2A) in IL-6 KO mice. Similarly, the amount of wheel-running activity was comparable between IL-6 KO and WT mice during periods of inactivity (rho) and activity (alpha) within the circadian cycle (Figures 2B–D). IL-6 deletion, however, was associated with an increased quantity of activity bouts (*p* < 0.05) with unchanged duration and amount of activity/bout (Figures 2E–G). Calculations of activity onsets and offsets revealed no differences between genotypes, and the duration of the active period was not statistically different between groups under LD conditions (Figure S1 in Supplementary Material).

**Figure 3:**
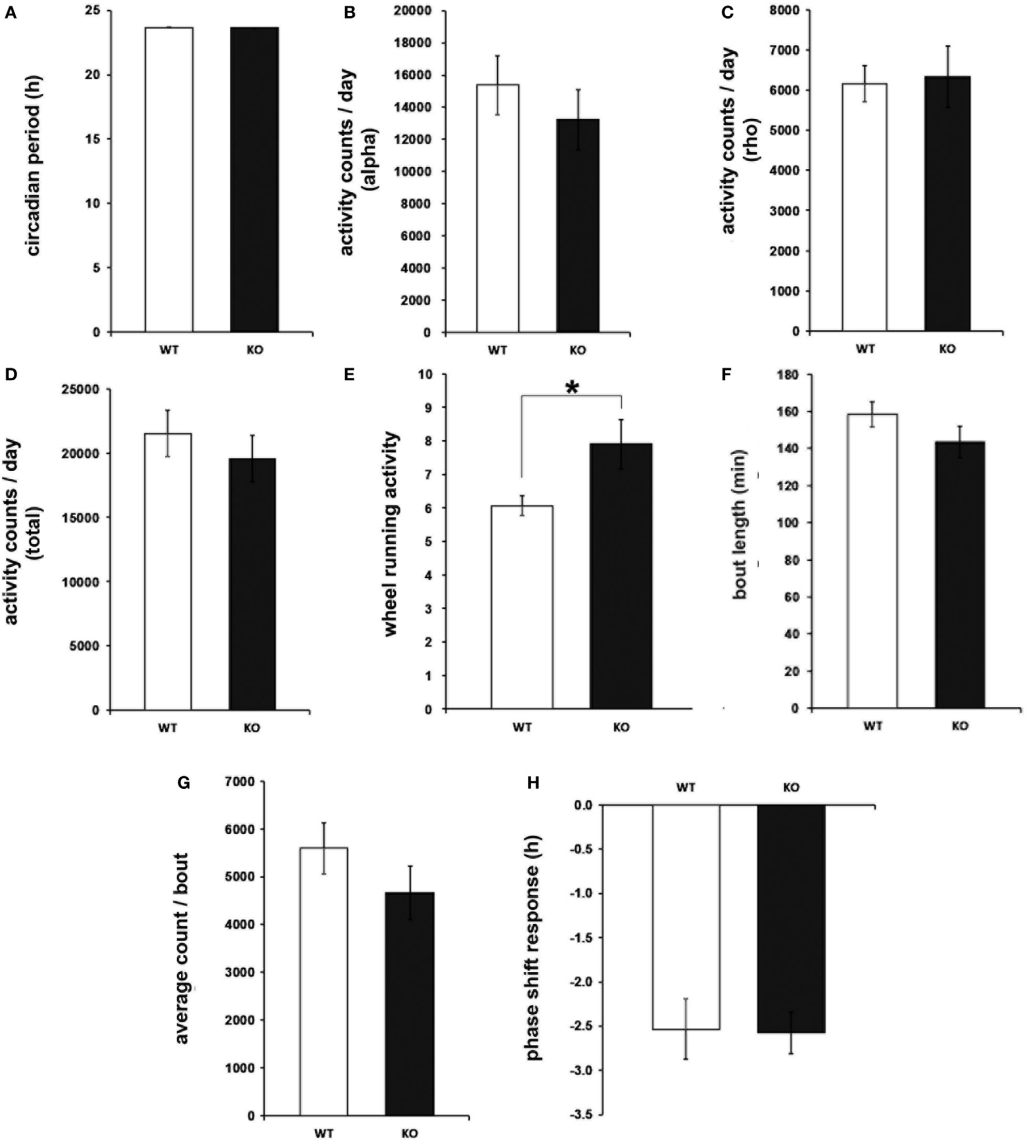
**Circadian period (tau), wheel-running activity, bout analysis, and phase shift response under free-running (dark/dark) conditions in interleukin (IL)-6 knockout (IL-6 KO) and wild-type (WT) mice**. Analysis of the free-running circadian behavioral locomotor activity in IL-6 KO and WT mice (*n* = 9–11 per group) demonstrating comparable **(A)** tau and wheel-running activity during the **(B)** alpha and **(C)** rho phase and in **(D)** total amounts. **(E)** Significantly increased quantity of activity bouts in IL-6 KO compared with WT mice with unaltered **(F)** bout length and **(G)** activity counts/bout. **(H)** Unaltered phase shift response to a brief light pulse at CT14 is in IL-6 KO mice. All data are displayed as mean ± SEM; *p < 0.05.

To determine circadian locomotor patterns during free-running rhythms, daily behavioral activity was further analyzed under DD conditions. In the same way as for the light-entrained rhythms, the circadian period, as well as the amount of wheel-running activity, was undistinguishable between IL-6 KO and WT mice (Figures 3A–D). Consistent with the results from the LD paradigm, the number of activity bouts was enhanced in IL-6 KO mice under DD conditions (*p* < 0.05), whereas no differences were seen in the duration and quantity of activity/bout or in the phase shift response in comparison with WT controls (Figures 3E–H). In addition, the duration of the active period was shorter in IL-6 KO mice under DD conditions (*p* < 0.05) (Figure S1 in Supplementary Material).

**Figure 4:**
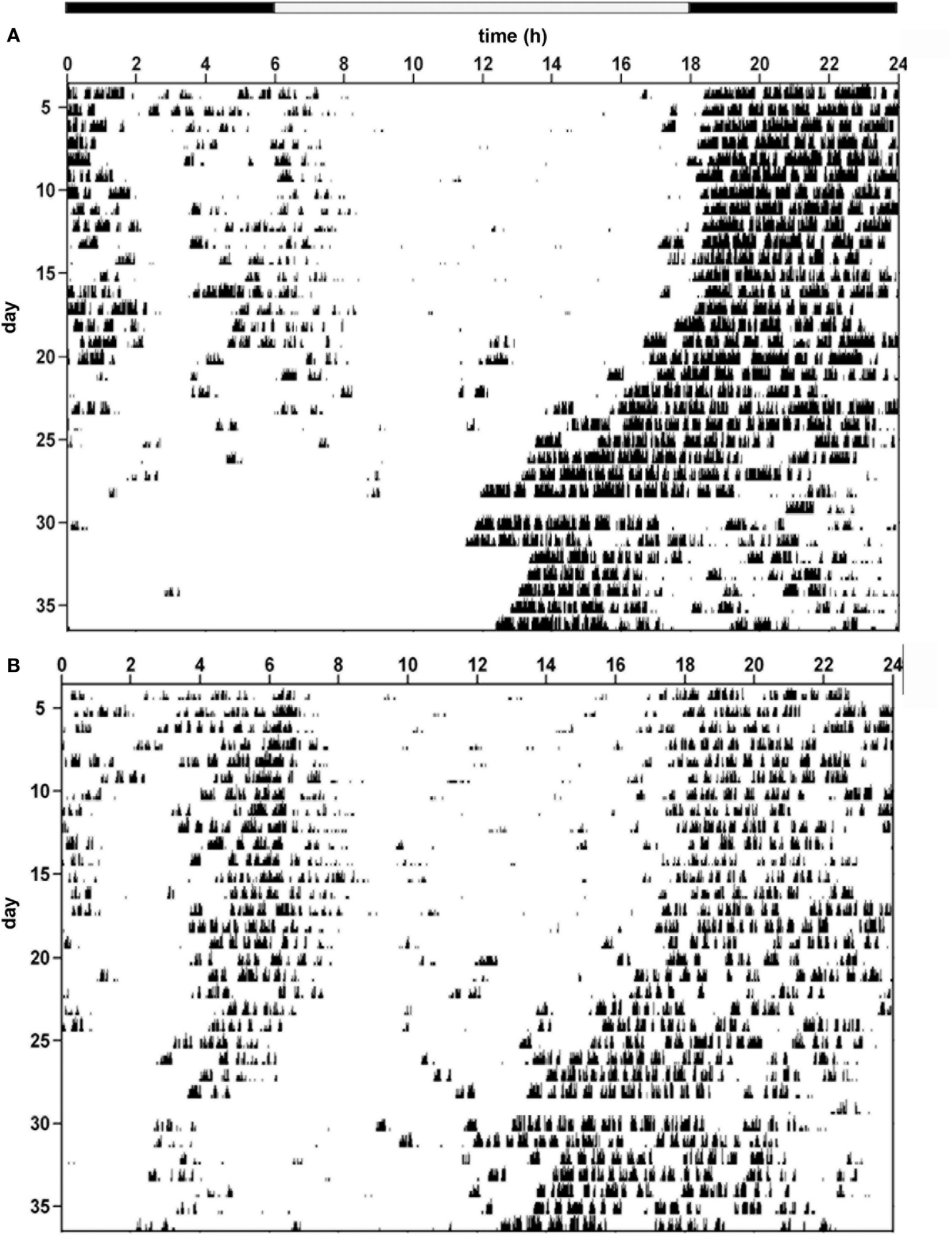
**Behavioral actograms exemplifying circadian locomotor activity patterns in interleukin (IL)-6 knockout (IL-6 KO) and wild-type (WT) mice**. Sample actograms illustrating wheel-running activity in **(A)** WT and **(B)** IL-6 KO mice.

Hence, the temporal architecture of the ultradian rhythms is disrupted in IL-6 KO mice under both LD and DD conditions as illustrated in the respective actograms of the two genotypes (Figures 4A,B). Further examples of representative actograms are provided in Figure S2 in Supplementary Material.

In addition, two-way ANOVA analysis (light condition × genotype) has been carried out to examine the possible effect of the light condition and its interaction with the genotype. The following main effects have been observed: for overall activity significant main effects of light condition for alpha: *F*_(3,43)_ = 88.54, *p* < 0.001 and rho: *F*_(3,43)_ = 178.17, *p* < 0.001. The characterization of the bouts revealed a significant main effect of genotype [*F*_(3,43)_ = 10.47, *p* < 0.01] for bouts per day and significant main effects of light condition for bout length: *F*_(3,43)_ = 29.98, *p* < 0.001 and counts/bout: *F*_(3,43)_ = 8.57, *p* < 0.01. The duration of the active periods revealed a significant main effect of genotype [*F*_(3,43)_ = 7.17, *p* < 0.05]. No other significant main effects or interactions were found.

### Aberrant mRNA Expression of Cry1, Dec2, and Rev-erb-Beta in the IL-6 KO Mouse Hippocampus

**Figure 5:**
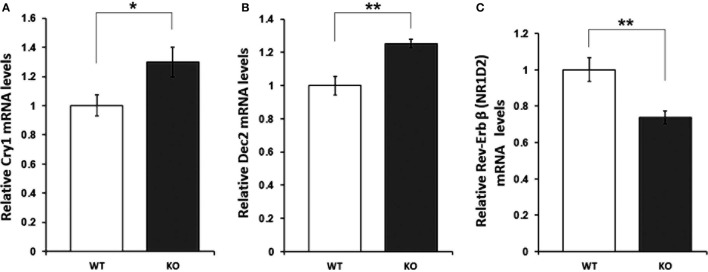
**mRNA levels of clock genes with significantly different expression in hippocampal tissue of interleukin (IL)-6 knockout (IL-6 KO) compared with wild-type (WT) mice**. Relative expression of **(A)** *cry1*, **(B)** *dec2*, and **(C)** *rev-erb beta* in hippocampal tissue of IL-6 KO compared with WT mice (*n* = 6–9 per group). All data are data displayed as mean ± SEM. *p < 0.05, **p < 0.01.

With regard to the molecular mediators of the observed alterations in the rhythmic oscillation of rest and activity patterns, mRNA levels of 19 clock (*clock, cry1/2, npas2, per1–3, rev-erb*α*/*β, and *ror*α*-*γ*)* and clock-controlled genes (dbp, e4bp4, id2, and neuroD1) were assessed in the hippocampus of IL-6 KO and WT mice. qRT-PCR analysis revealed a significant increase in levels of *cry1* (*p* < 0.05) and *dec2* (*p* < 0.01), whereas expression of *rev-erb-beta* (*p* < 0.01) was reduced in IL-6 KO compared with WT controls (Figure 5). No differences in the mRNA of any of the other clock genes investigated were found (Table 1).

**Table 1:** **Clock and clock-controlled genes with comparable mRNA levels in hippocampal tissue of knockout (KO) and wild-type (WT) mice**.

Gene name	WT (rel. expression)	KO (rel. expression)	*p* Value
*clock*	1.000 ± 0.1293	1.0197 ± 0.0038	0.6
*cry2*	1.000 ± 0.1332	0.9949 ± 0.0168	0.9
*dbp*	1.000 ± 0.0916	1.0272 ± 0.0140	0.4
*dec1*	1.000 ± 0.1414	0.9893 ± 0.0406	0.8
*e4bp4*	1.000 ± 0.1375	0.9952 ± 0.0084	0.8
*id2*	1.000 ± 0.0902	1.0385 ± 0.0371	0.4
*neuroD1*	1.000 ± 0.0673	1.0045 ± 0.0038	0.7
*npas2*	1.000 ± 0.0759	0.9993 ± 0.0192	0.9
*per1*	1.000 ± 0.0841	1.0516 ± 0.0468	0.3
*per2*	1.000 ± 0.1055	1.0291 ± 0.0105	0.2
*per3*	1.000 ± 0.2047	1.0170 ± 0.0206	0.7
*rev-erb*α*/*β	1.000 ± 0.0740	1.0367 ± 0.0181	0.2
*ror-*α	1.000 ± 0.1685	0.9714 ± 0.0344	0.5
*ror-*β	1.000 ± 0.0731	0.9714 ± 0.0191	0.2
*ror-*γ	1.000 ± 0.1034	0.9829 ± 0.0092	0.2
*bmal1*	1.000 ± 0.1180	1.0225 ± 0.0131	0.3

*Fold change values in KO mice (normalized to WT means for each transcript) of clock and clock-controlled (gray) genes are displayed as mean ± SEM (*n* = 6–9 per group). *p* Values represent results of statistical analyses using two-tailed Student’s *t*-tests*.

## Discussion

Most species living on the surface of earth have evolved under conditions of rhythmically changing daily variations in fundamental environmental constituents, such as light. To anticipate and respond to these oscillating physical properties, organisms have developed systems to accordingly fit their physiology. Hence, the most essential functions of the body, including those of the nervous and the immune systems, are determined by these intrinsic timing regulations. Thus, the association between disruption in “biological clocks” and pathologies of the brain (31, 51–53) and the immune response is unsurprising [see for review Ref. (54)]. Indeed, the circadian regulation of the behavioral states of activity/rest (as fundamental output of brain function) is well described. Similarly, evidence for the impact of the endogenous clockwork on the most pivotal elements of the body’s defense mechanisms, such as the release of immune modulatory substances, is augmenting (55–58) [see for review Ref. (59)].

The current report is, to the best of our knowledge, the first comprehensive, long-term assessment of the impact of a genetic deficiency in a central element of the immune response (the proinflammatory cytokine IL-6) on circadian wheel-running activity rhythms in the mouse. This interrelationship is particularly noteworthy within the framework of diseases and disorders in which all these functions are of pathophysiological relevance, as is the case for the neurodegenerative AD and the neuropsychiatric MDD, where the involvement of the circadian and the immune systems have been extensively demonstrated (31). In the case of both these mental illnesses, frequent presentations of aberrant diurnal oscillations of behavioral activity have been reported in patients and in subjects of the respective experimental animal models (15–29, 31, 60–62).

In the herein studied IL-6 KO mice, traditional parameters of diurnal behavioral rhythmicity were unaltered under light-entrained and free-running conditions, as tau and the amount of activity during active and inactive phase were comparable with those of WT controls but were determined by the light conditions (LD versus DD) for both genotypes. Interestingly, the duration of the active period was shortened in IL-6 KO mice. In a previous short-term evaluation of home cage behavior, higher activity of IL-6 KO compared with WT mice has been reported (63). However, the analysis of home cage activity does characterize a behavioral output distinct from circadian wheel-running activity (64). Although home cage activity reflects the baseline activity, wheel running is an elective action, which is driven by additional endogenous factors, such as motivation (64). However, it is the only system to reliably address some distinct features of the internal timekeeping system, such as the modulation of the endogenous circadian machinery by environmental stimuli. Indeed, an unaltered phase-shift response in IL-6 KO mice indicated an intact responsivity of the endogenous CT keeping system to an external *zeitgeber*. Hence, the 24-h structure of the behavioral locomotor rhythm seemed largely preserved IL-6 KO mice. However, a close examination of the activity bouts as indicators of units of ultradian activity revealed a significant difference in the number of bouts between genotypes, independent of the external lighting conditions: IL-6 KO mice presented with an augmentation in the number of bouts/circadian day, while the bout length and activity/bout remained unchanged. This result is also reflected in the two-way ANOVA analysis, which revealed a significant main effect of genotype for the number of bouts, whereas interestingly the bout length and activity/bout were significantly dependent on the light conditions for both WT and KO mice.

The nature and regulation of ultradian rhythms and activity bouts is less well described than is the case for the classical indicators of diurnal rhythms, e.g., length of the circadian period tau and activity onsets and offsets, which are largely dependent on the suprachiasmatic nucleus (SCN) of the hypothalamus as a central circadian pacemaker (65–70). The SCN also orchestrates rhythmic activities in other regions of the brain and peripheral parts of the body with synchronization of clock gene expression as a pivotal molecular event.

To examine potential neurobiological mechanisms contributing to the observed phenotype of IL-6 KO mice, we decided to focus on the hippocampus, a brain region involved in the pathophysiology of AD (71–73) and MDD (74, 75). Examination of the expression of major clock genes as molecular mediators of circadian rhythmicity revealed a selective effect of genetic IL-6 deficiency on the hippocampal mRNA levels of *cry1, dec2*, and *rev-erb-beta*.

Although the statistically significant expressional differences between IL-6 KO and WT mice were modest in magnitude, they may be well of biological relevance considering the role of these genes in the tightly controlled feedback loops of transcription–translation from which circadian rhythms are generated at the molecular level (20, 24, 28, 76). The increased levels of *cry1* in IL-6 KO are paralleling observations in plasma levels of sepsis patients were an increase in IL-6 was associated with a decrease in *cry1* mRNA (77). A modulatory influence of several immune mediators on the expression of *dec2*, which is here to be reported significantly reduced in the hippocampal tissue of IL-6 KO mice, has been described. Interestingly, IL-6 is a direct activator of AMP-activated protein kinase (78), which has been found to mediate the regulatory effects of *dec2* in several tissues (79).

Previous work reports that *rev-erb* expression in peripheral blood leukocytes of human subjects, together with several other clock genes (including *cry1*), is dampened by endotoxin treatment, which leads to a concomitant increase in circulating levels of IL-6. This description is in line with our observation on augmented *rev-erb-beta* and *cry1* levels in IL-6 KO.

Alternatively or additionally to a mechanistic involvement of clock gene expression, the alteration in the ultradian architecture of behavioral activity in IL-6 KO mice may relate to the direct regulatory effect of IL-6 on the serotonin transporter (SERT) (80). Indeed, multifaceted interactions between the circadian and the serotonergic systems have been demonstrated with a proposed role of these interrelationships for several mental illnesses, including MDD [see for review Ref. (81, 82)]. However, although a defined role for dopamine and the dopamine transporter in the regulation of ultradian rhythms of locomotor behavior have been proposed (83), a potential involvement of SERT in the control of ultradian activity architecture remains to be examined in future studies.

Some conceptual restrictions, which were imposed by the study design, such as the determination of clock gene expression at a single time of the day in *a priori* selected brain region of interest have to be considered for the interpretation of the results obtained. Hence, the observed differences in clock gene expression between IL-6 KO and WT mice do not allow for conclusions regarding the diurnal oscillation in the expression of these genes in the two genotypes, an important mechanistic insight that will be addressed in follow-up investigations. Within this framework, however, this study allows for the deduction of three major conclusions: first, IL-6 is not required for diurnal time keeping of the circadian period under either light-entrained or free-running conditions; second, genetic IL-6 deficiency is associated with aberrant ultradian activity patterns as reflected in an increased number of activity bouts with unaltered length and activity counts per bout, independent of the external light conditions; and third, a selective modulation of hippocampal clock gene expression proposes an involvement of disrupted mRNA levels of *cry1, dec2*, and *rev-erb-beta* in the circadian phenotype of IL-6 KO mice.

Collectively these data suggest a potential pathophysiological involvement of the pro-inflammatory cytokine IL-6 in the circadian alterations associated with severe neurological and psychiatric disorders and invite further investigations on the underlying molecular mechanisms.

## Author Contributions

FM co-designed experiments, analyzed data, and co-wrote the manuscript; AC and JA analyzed data; IE and OH conducted behavioral experiments; WD carried out gene expression analysis; and DP and MG conceived the study, analyzed data, and wrote the manuscript.

## Conflict of Interest Statement

The authors declare that the research was conducted in the absence of any commercial or financial relationships that could be construed as a potential conflict of interest.

## Supplementary Material

Figure S1 **Duration of the active period (*alpha*) and activity onsets and offsets in interleukin-6 (IL-6) and wild-type (WT) mice**. Analysis of the length of the active period in IL-6 and WT mice (*n* = 9–11 per group) under **(A)** light/dark and **(B)** dark/dark conditions. **(C)** Activity onsets and **(D)** offsets in circadian hours in IL-6 compared with WT mice. All data are displayed as mean ± SEM; **p* < 0.05.

Figure S2 **Behavioral actograms exemplifying circadian locomotor activity patterns in interleukin-6 (IL-6) and wild-type (WT) mice**. Sample actograms illustrating wheel-running activity in **(A)** WT and **(B)** IL-6 mice.
