# Supplementary figures and images for: Disrupted Ultradian Activity Rhythms and Differential Expression of Several Clock Genes in Interleukin-6-Deficient Mice
Source: Front Neurol. 2017 Mar 22;8:99. doi: 10.3389/fneur.2017.00099 (PMC5360714; doi:10.3389/fneur.2017.00099)

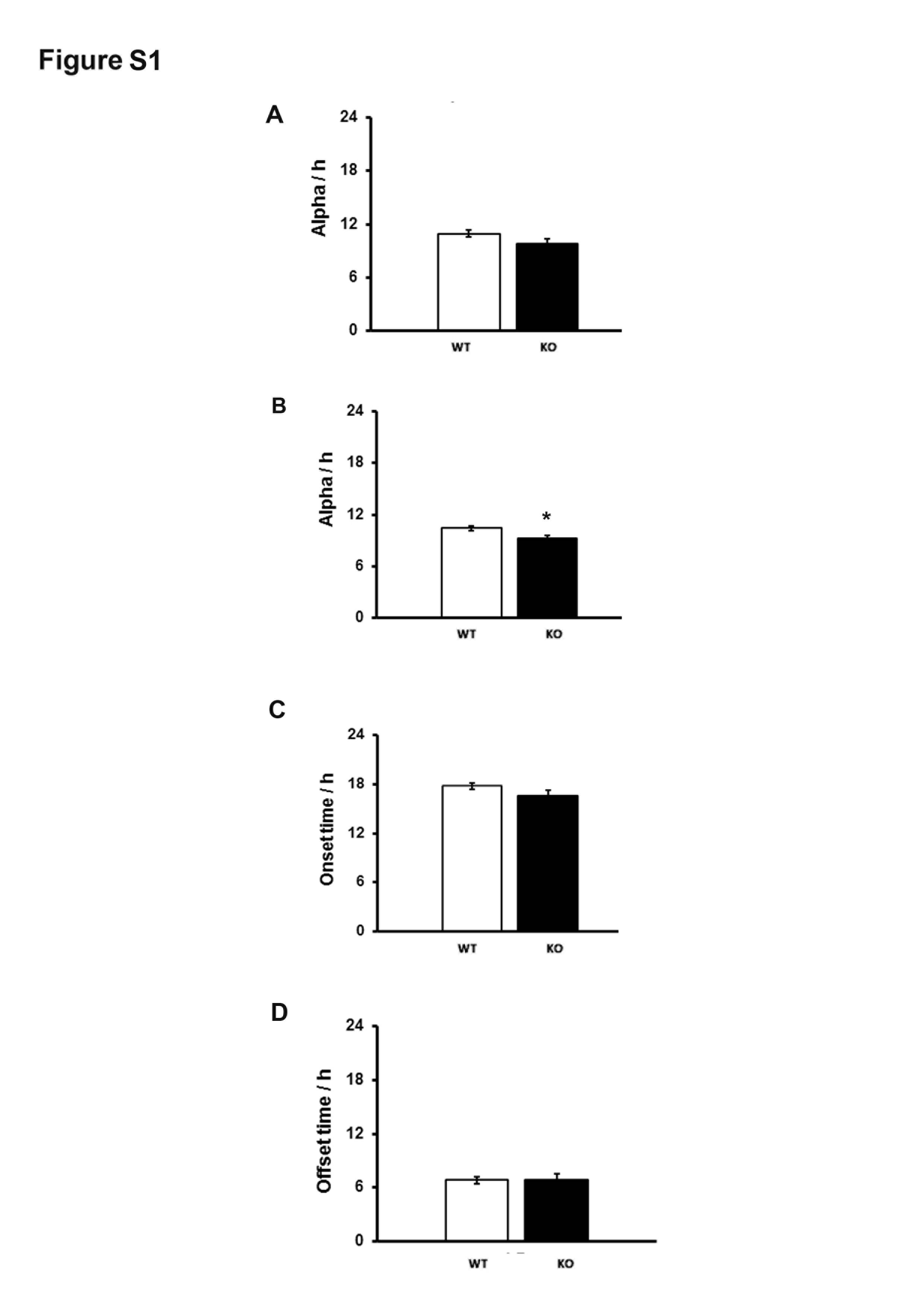

Supplement: Figure S1 — Duration of the active period (alpha) and activity onsets and offsets in interleukin-6 (IL-6) and wild-type (WT) mice. Analysis of the length of the active period in IL-6 and WT mice (n = 9–11 per group) under (A) light/dark and (B) dark/dark conditions. (C) Activity onsets and (D) offsets in circadian hours in IL-6 compared with WT mice. All data are displayed as mean ± SEM; *p < 0.05. [file Image_1.TIF]

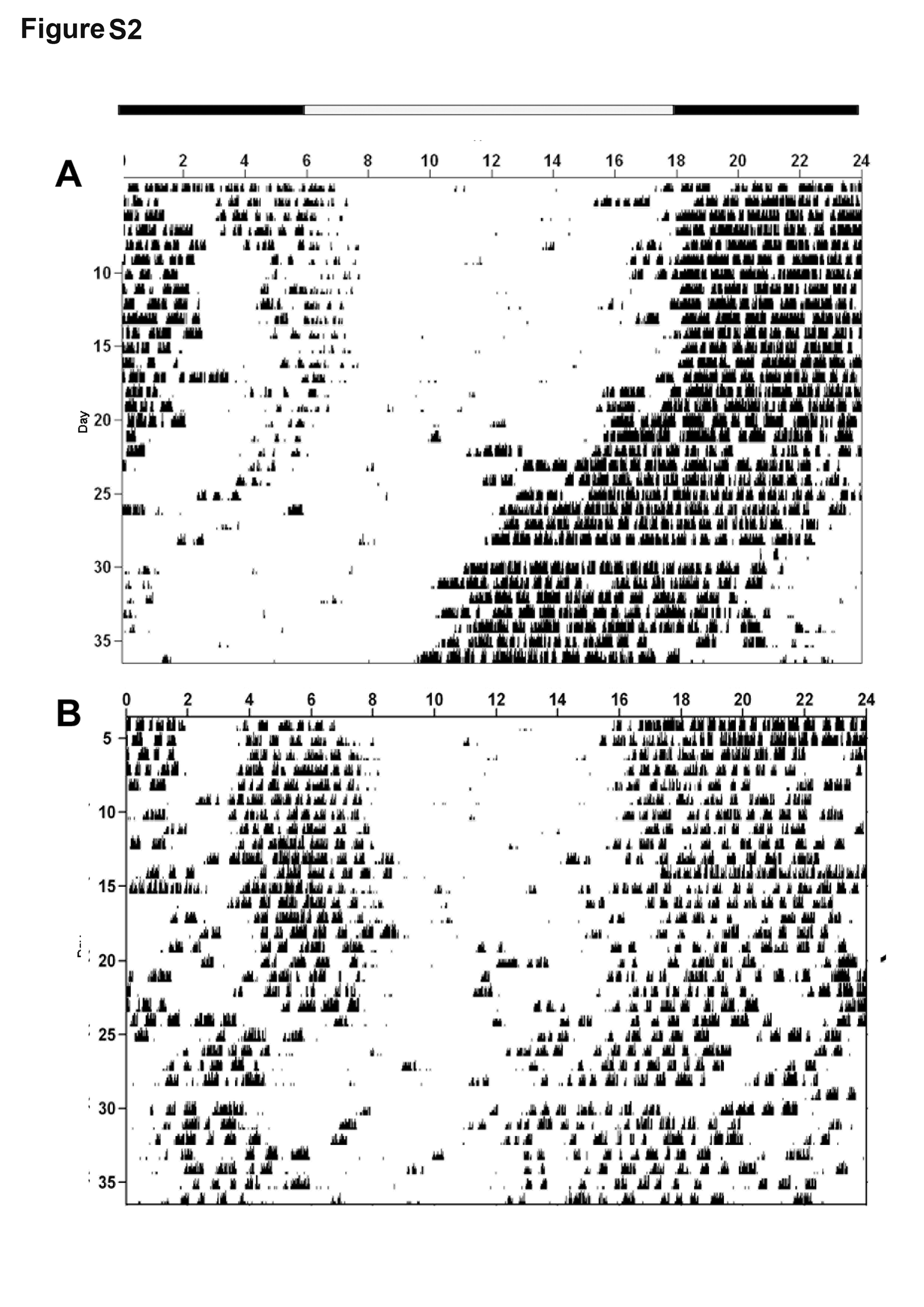

Supplement: Figure S2 — Behavioral actograms exemplifying circadian locomotor activity patterns in interleukin-6 (IL-6) and wild-type (WT) mice. Sample actograms illustrating wheel-running activity in (A) WT and (B) IL-6 mice. [file Image_2.TIF]
